# Supplementary material for: Patterns of Intron Gain and Loss in Fungi
Source: PLoS Biol. 2004 Nov 30;2(12):e422. doi: 10.1371/journal.pbio.0020422 (PMC532390; doi:10.1371/journal.pbio.0020422)
Supplement: Table S1 — Also available at http://genes.mit.edu/NielsenEtAl/. (4.3 MB ZIP). [file pbio.0020422.st001.zip › NielsenEtAl/html/1265.html]

AN2477.1.NCU08273.1.MG01769.1.FG09753.1


```
 CLUSTAL W (1.82) Multiple Sequence Alignments - Introns Inserted


Sequence 1: NCU08273.1	758 aa
Sequence 2: MG01769.1	752 aa
Sequence 3: FG09753.1	737 aa
Sequence 4: AN2477.1	841 aa
Alignment Length: 888 aa
Number Identitical Residues: 266 aa
Alignment Score (without introns) 15213


MG01769.1 	------------------------------------~-----------------------
NCU08273.1	------------------------------------~-----------------------
FG09753.1 	------------------------------------~-----------------------
AN2477.1  	MGPSTMSPNCRHTVQIKTTLTEHVPFGRYGVLGSTV1QQITILRQAHEGPKLAKLRVQLG
          	 ..:: :...  : . .:: :.  . .  .  .::  .. :   .: .... :.   . .

MG01769.1 	-MATLKTLY------------------------------------GSDSVSRPELNENFG
NCU08273.1	-MADPLSFY------------------------------------LEDDKTQPGTRSNFN
FG09753.1 	-MSNLKDLY------------------------------------GQSG--DKSQTSNFG
AN2477.1  	RMTTFKVLISFISIAGPLGVGWFFLDSPLSDLLLPKPKLGFDQIIEMASSALSGSHNNFY
          	 *:    : :  : :.. . .    .:. :.   .... . ..     . :     .** 

MG01769.1 	2VDYVVDYHIPPSE~---------------------AERAEAEAIFIQLVKALAAVGFAT
NCU08273.1	2VDYVIHYKLPTTS~GLSAPGMASLAASDDTNIRPVKEKEEAEAAFIQLINALSAVGFAT
FG09753.1 	2VDFVIHYKVPPSE1-----------------------RAEAEAGFVQLIQALTTVGLAT
AN2477.1  	~ADYVIQYNYANTE1P-----------------------TKASEELELLLRKLWEVGLQA
          	 .*:*:.*: . :.                          :*.  :  *:. *  **: :

MG01769.1 	EVRHGGKSSLLIFLKITSDKLLHAQVYRHR~LQDWLYGVRASAPNKQDLTNHFEEEPVSE
NCU08273.1	EVRPGGKSSLLVFTKIASEKLLTSQVYRYR~IQDWLFGVRTSAPN-QDLSKYLEEEPVSE
FG09753.1 	EVRHGDSDSLLVFVKIASPDLFAKQVYRAR~LGDWLHGVRVSAPH-NDVNQALEDEPVVE
AN2477.1  	EVRPGEDSNLLVFVRASRKKSLRRAIYQSR2IRDWLHGVRNSEPE-----DESSAEPQTE
          	*** * ...**:* : :  . :   :*: * : ***.*** * *.     .  . **  *

MG01769.1 	AERLRLAYMLICKPKNEGGAGITPRSGQWKHVASVFPLHDHRFNRDWIKRWSTKYILDDA
NCU08273.1	AERLRLTYLLITKSKNEGGAGITAKVGKWKYVDSIFPLHDHDFNRRWIKAWSSKYFLDEE
FG09753.1 	AERLRLIYLMITKPRDEGGAGITTTNSNWKYVDSVFPLHSHNFNKQWIKKWSSKSTLEQA
AN2477.1  	SERLRIINHMITLPHNVGGAGITPKHGHWKNVLAVFPLHDEEKNREYLADWSKKTFLSEE
          	:****:   :*  .:: ******.  .:** * ::****..  *: ::  **.*  *.: 

MG01769.1 	DLNEIRDKFGEN~VAFYFAFLQSYFKFLVFPAAFGFAAWLVLGKFSWVYALVNCLWSVVF
NCU08273.1	DLNRIRDKFGER~VAFYFAFLQSYFAFLLFPAAFGFAAWLILGKFSWFYAVVNALWSVVF
FG09753.1 	DIDEIRDKFGES~VAFYFAFLRSYFRFLVFPAGFGFVSWLLLGQFSFLYALLCGLWSVVF
AN2477.1  	DLDQIRGKFGES0VGFYFAFLQSYFKFLLFPAVFGFSCWLLLGSFSIIYTVVNCLWGVIF
          	*::.**.****  *.******:*** **:*** *** .**:**.** .*:::  **.*:*

MG01769.1 	FEYWKKKEVDLAVQWGVRGVSKIQQPRPQFQHESEAQDPVTGEIIKVFPPLKRLSRQLLQ
NCU08273.1	FEHWKTKEVDLAVRWGVRGVSRIQHPRPQFQFEREAQDPVTGEIVRVYSPFKRLARQLLQ
FG09753.1 	FEYWKKKEIDLAVQWGVRGVSSIQQARPEFEWDREAEDPVTGEAVKVYEPMKRIKTQLLQ
AN2477.1  	IEYWKRQEEDLSCRWQTKGVSVVRSKRREFKPEREVYDQVTGETRGVFPATTRLQRQLLQ
          	:*:** :* **: :* .:*** ::  * :*: : *. * ****   *: . .*:  ****

MG01769.1 	IPFAIACMGVLGSLIFSCFAIEIFITEVYNGPFKQYL~TFLPTVLLTIFMPALTALLTSL
NCU08273.1	IPFALACVVVLGGLIVSCFSIEVFITEIYTGPFKQYL~TFLPTVLLTIFMPTFSGLLTNL
FG09753.1 	IPFALAAVIALGALIVTCNSLEVFINEVYTGPGKQYL0AFLPTVFLVLGTPTISGLLMSA
AN2477.1  	VPFALVALVALGAIIATCFAIEIFISEVYTGPLKSYL0VFIPTILLSALIPTMSGVLVSI
          	:***:..: .**.:* :* ::*:**.*:*.** *.** .*:**::*    *:::.:* . 

MG01769.1 	ADKLTALENYETEAA~HHASFVQKMFVINFITAYMPLFLTAFVYIPFGKVLAPHLDVFQV
NCU08273.1	AEKLTKAENYETHDA~HQASFIEKIFVLNFITSYLPIFLTAFVYVPFGKLLVPYLDVFKV
FG09753.1 	AEKLNSMENYATVDA1HDAALIQKQFVLNFMTSYMALFFTAFVYIPFGHVLVPFLEFWRK
AN2477.1  	ATKLNDYENYETQTA~YDTALTQKIFVINFITSYFPILLTAFVYIPFASLIVPHLDVFHL
          	* **.  *** *  * :.::: :* **:**:*:*:.:::*****:**. ::.*.*:.:: 

MG01769.1 	TARKLTKEGKGIK-TKEFHVNPDRFSKQMFYFTVTAQIVNLLLEVFVPYFKRKVFKAVEK
NCU08273.1	TAQSFTTMEKPV--TKSWEINPDRLTTQVIYFTVTAQIVNFLTEVIVPYAKRKVFKKVQE
FG09753.1 	TAQVVTFSDKELP-TQNFQINPARISNQMFYFTVTAQIVNFATEVVVPYVKREVFQKAKE
AN2477.1  	TVRPFVSKEKATTAHENFTINPDRLRKQVIYFTVTAQVVGFAMETIVPFLKQRFAREYKE
          	*.: ..   *   : :.: :** *: .*::*******:*.:  *..**: *:.. :  ::

MG01769.1 	VQDG-----------IHHSG-TVHHQDSPEEAAFLGRVRDEAMLEVYDVTTDYREM1VVQ
NCU08273.1	VQSE-----------FTGNGHELQIKDHPDEADFLKRVRNEAELDAYDVTVDYREM~VVQ
FG09753.1 	LKSK-----------PKIQD------DHEEEAEFLERVRKECTLEVYDVSGDYREM~VMQ
AN2477.1  	YKKKKYGRVNSGLVSETQKETQHSYDDHEDEVQFLTRVRKEADLDDYNVTDDLREM~CIQ
          	 :. . .  .:.  :   .      .*  :*. ** ***.*. *: *:*: * ***  :*

MG01769.1 	FG~YLSLFSVAFPLTACSFLINNWVEARSDAMKIAIGSQRPIPWRNDTIGPWLTSLGFLS
NCU08273.1	FG~YLSLFSVIWPLAACSFLVNNWVEARSDAMKIAIGSQRPVPWRADSIGPWLNSLGFLS
FG09753.1 	FG1YVAMFSVAWPLAACCYLVNNWVELRSDALKIAISSRRPIPWRTDSIGPWLTALSFLS
AN2477.1  	FG~YLALFSPVWPLVPVSFLVNNWVELRSDFFKIIVECKRPTPIRVDTIGPWLDTLGFLS
          	** *:::**  :**.. .:*:***** *** :** : .:** * * *:***** :*.***

MG01769.1 	WLGSLTSAAVVYLFSSGNDRQGPDGRPSNLSGWALLLSIFVAEHVYLVVQLAVRYVVEKL
NCU08273.1	WLGSITSAALVYLFN--KHDINPDGTPWDISGWALLLSILFAEHIYFAVQLVVRSLVQKL
FG09753.1 	WLGSITSASIVYLCS-GARGHGVHGTPSPLKAWGLLLSILLAEHFYLVVQLAVRYVLSKL
AN2477.1  	WVGSITSAALVYMFN--TSTNGPNGEPSAIKGWALLLTIFFAEHLYLIVRYVVQATIAKF
          	*:**:***::**: .      . .* *  :..*.***:*:.***.*: *: .*:  : *:

MG01769.1 	DSPGLQKERAERFALRKRLLDDTLGHD------EAEKAATAVLSGSEKITRDSLEEEARI
NCU08273.1	DNPGLQKERAERYALRKKLLEQTFGSHGYDADLTEEARGPGYVSSGERITREALEEEARV
FG09753.1 	DSPGEQKERRERFMTKRKLLEENIGQG------ASEKASVPGIDHSEKITRAALEEEARQ
AN2477.1  	EPHNVRREKTEQYMIRKRYLEATLGAR--SSDEEEEVTAKDESMELSDLTRKSLEDDTRA
          	:  . ::*: *::  ::: *: .:*    .:.   *  .       . :** :**:::* 

MG01769.1 	ASTKGHGSPEES2FWLRQRGAGETIEVGRRLISE0V~ESAHNATKTVH1ALAVDRGGCEV
NCU08273.1	NATK---TPEQL~FWLRQRGAAETIEIGRGLMAE~V~STNLSLTS---~-----------
FG09753.1 	TSIRGHGTPEEM2FWQRQRGMNETIDVGRRMIEQ~Q0AAAQKNGQSKS~PVPSMKAS---
AN2477.1  	WSKHN-TVAEER~FWMRQKGWRESAQVAASIIES~Q~AKPDMKKQQ--~-----------
          	 : :.   .*:  ** **:*  *: ::.  :: .          .               

MG01769.1 	G
NCU08273.1	-
FG09753.1 	-
AN2477.1  	-
          	
```
